# EGFRvIII expression in head and neck squamous cell carcinoma: clinical significance and sources of frequency variation across studies

**DOI:** 10.3389/fonc.2026.1790296

**Published:** 2026-02-25

**Authors:** Sherin James, Benjamin B. Kasten, Jackie Shi, Akhilesh M. Wodeyar, Julian Barnhill, Ivan Reap, Carissa M. Thomas, Anthony B. Morlandt, Eben L. Rosenthal, Jason M. Warram

**Affiliations:** 1 Department of Otolaryngology, University of Alabama at Birmingham, Birmingham, AL, United States; 2 Department of Biology, University of Alabama, Tuscaloosa, AL, United States; 3 Department of Otolaryngology-Head and Neck Surgery, University of Colorado, Aurora, CO, United States; 4 Department of Oral and Maxillofacial Surgery, University of Alabama at Birmingham, Birmingham, AL, United States; 5 Department of Otolaryngology-Head and Neck Surgery, Vanderbilt University, Nashville, TN, United States

**Keywords:** biomarker, clinical significance, detection methods, EGFRvIII, frequency variation, head and neck squamous cell carcinoma, therapeutic target

## Abstract

EGFRvIII is a tumor-specific, gain-of-function mutation of the EGFR gene that was first detected in 1990 in glioblastoma. For the past two decades, its significance in head and neck cancer has been intensely debated, both in terms of its clinical implications and its mere presence in the disease. This review aims to synthesize evidence on the prevalence, frequency, detection methods, and clinical significance of EGFRvIII in head and neck cancer studies. Our search included major databases such as PubMed, Embase, and Web of Science with keywords such as EGFRvIII, EGFR variants, and head and neck cancer, and stratified the results using Boolean logic to enhance relevance and specificity. Data extraction involved classifying studies by detection method, anatomic subsite, etiology, geography, and population size. The results revealed a frequency of EGFRvIII expression ranging from 0 to 75% across studies, with the major factors influencing this variation being technical sensitivity and specificity issues, primer set variability, sample type and quality heterogeneity, low prevalence and statistical power, and the lack of validation standards. There appears to be an association with a poorer clinical prognosis, though the association with survival remains inconsistent across studies. For future research, it is preferable to be informed about methodological rigor and orthogonal diagnostic assays with extensive prospective validation. Understanding EGFRvIII’s genuine frequency and prognostic utility in HNSCC will help guide biomarker development and targeted therapies.

## 1 Introduction

The discovery of epidermal growth factor (EGF) in the early 1950’s by Levi-Montalcini and Cohen provided first answers to the fundamental question of how cells recognize and respond to external growth signals. This was further elucidated in the mid1970’s with the identification of a plasma membrane protein that binds EGF - the epidermal growth factor receptor (EGFR/Her1/ErbB1), a 170 kDa, membrane-spanning glycoprotein belonging to the receptor tyrosine kinase (RTK) family of growth factor receptors (1, 2). Mutations in the epidermal growth factor receptor occur frequently in a number of human cancers, including gliomas, non-small-cell lung carcinomas, breast and ovarian adenocarcinomas, prostate, and head and neck carcinomas (HNC) (1, 3). A commonly described EGFR mutation is type III (variously named EGFRvIII, truncated EGFR, de2–7 EGFR, EGFR^vIII,^ or ΔEGFR), which lacks a portion of the extracellular ligand-binding domain (4).

**Figure 1:**
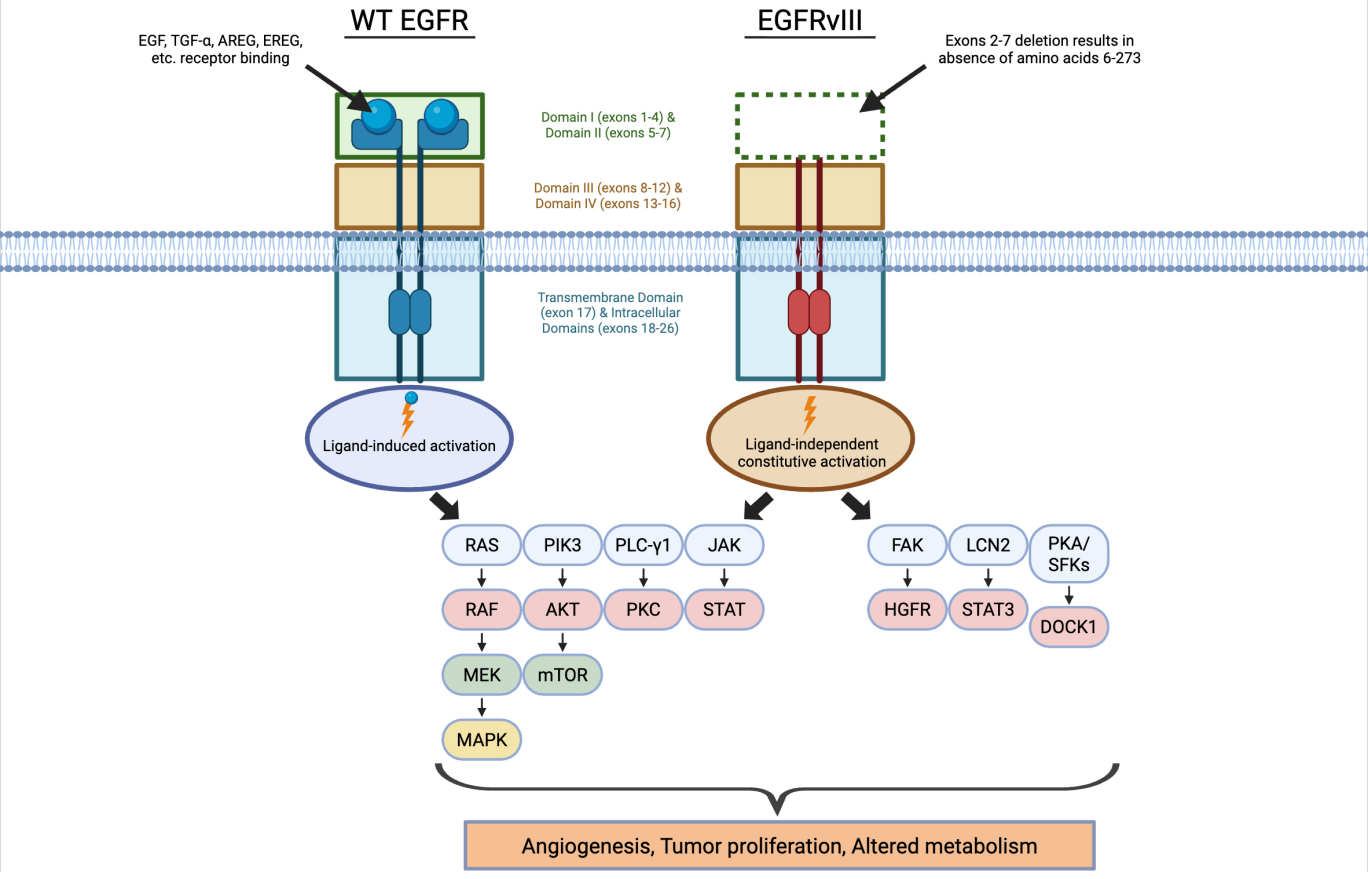
Molecular architecture and signaling differences between WT EGFR and EGFRvIII in HNSCC. WT EGFR is activated by the binding of various ligands whereas EGFRvIII exhibits truncations in its extracellular domain and is constitutively active. Both WT EGFR and EGFRvIII promote tumorigenesis via pathways initiated by the phosphorylation of certain effector proteins and their respective pathways. Additionally EGFRvIII exhibits unique downstream effectors separate from WT EGFR.

EGFRvIII is a tumor-specific, extracellular, gain-of-function mutation of the EGFR gene detected in 1990 (5). It is commonly studied and characterized in glioblastoma (GBM) (6), where it is frequently found with EGFR amplification and is associated with enhanced proliferation, tumor growth, and resistance to therapies such as radiation and targeted therapy (cetuximab) (7–11). This mutation results from an intragenic in-frame deletion of exons 2–7 (267 AA) of the EGFR gene, creating a truncated, constitutively active receptor not bound by ligand (12) (**Figure 1**). The deletion removes ~801 bp, including almost all of domain I and half of domain II of the extracellular region, making the mutated protein (145 kDa) have a unique glycine codon at the exon 1–8 junction (13, 14). In contrast to wild-type (WT) EGFR, the deletion in EGFRvIII results in constitutive kinase signaling at relatively low levels. This ligand-independent activity acquires biological significance via resistance to degradation through increased membrane stability (3, 15). The mutant can form both homodimers and heterodimers with other receptors, potentially activating multiple signaling pathways even in the absence of ligands. Even though the receptor is constitutively active and exhibits less efficient internalization and prolonged surface retention, the oncogenic potential of the oncogene primarily stems from its exceptional membrane stability rather than its kinase activity (3). Free cysteine in EGFRvIII’s extracellular domain (due to deletion) allow disulfide-linked dimers/oligomers, which are important for signaling.

Since EGFRvIII expression is exclusive to tumor cells and absent in normal tissue, it presents as an ideal therapeutic target for exploitation, despite incomplete understanding of its oncogenic role (10). In HNC, WT EGFR is overexpressed in 80 - 90% cases (16–18) and EGFRvIII is said to be expressed in a wide range of frequency from 0 to 75%, i.e. from not expressed (19) to very low (20) to high (21, 22), which is a dramatic distribution requiring further examination (7, 12, 19, 20, 22–27). Publications related to EGFR targeted therapies surged in the late 2000s to early 2010s due to clinical validation of EGFR as a pharmacologically targetable oncogene shown by the approval of cetuximab in 2006 (28). The following emergence of secondary resistance mutations and activation of alternative pathways, which limited the efficacy of EGFR-targeted therapy, led to the development of second and third-generation EGFR inhibitors (29). However, these targeted therapies still show limited benefit in some patients (30), and the presence of EGFRvIII was theorized to explain the observed resistance and tumor heterogeneity. The true clinical impact of EGFRvIII in HNSCC is in question. Hence the following sections will review reported EGFRvIII frequencies in HNSCC, dissect methodological and biological contributors to variability, and propose a path toward standardized detection and clinical implementation to pave the way for robust biomarker‐driven trials.

## 2 Molecular mechanism

The general mechanism of EGFRvIII in HNSCC is different from GBM. Unlike GBM, HNSCC tumors exhibit co-expression of EGFRvIII with WT EGFR and express EGFRvIII in tumors with or without EGFR amplification (26). Multiple mechanisms like genomic deletion, intron rearrangements, and regulatory factors contribute to EGFRvIII expression in HNSCC (26). WT EGFR activates RAS–RAF–MEK–MAPK, PI3K/AKT, and JAK/STAT pathways, enhancing tumorigenic potential (31) (**Figure 1**). By comparison, EGFRvIII in HNC drives enhanced oncogenicity through selective activation of Src family kinases most notably Lyn, resulting in persistent PI3K/Akt, MAPK and STAT3 signaling which drives tumor-intrinsic aggressiveness like proliferation, migration, invasion, and MMP-mediated ECM remodeling (22, 23, 32) (**Figure 1**; **Table 1**). The downstream effects of EGFRvIII-expressing cells secrete IL-6 and LIF (leukemia inhibitory factor), and these cytokines activate gp130 signaling in neighboring cells (33) and contribute to a non-cell-autonomous effect of EGFRvIII signaling via autocrine and paracrine signaling. The tumorigenic signaling amplifies and diversifies EGFRvIII oncogenic signaling and interacts with other receptor tyrosine kinases (RTKs), suggesting it acts not only as an autonomous receptor but also as a complex signaling hub (34). EGFRvIII forms ligand-independent dimers, including heterodimers with WT EGFR and other RTKs, leading to trans-phosphorylation of WT EGFR. The heterodimer potency seems to be greater than homodimers of EGFRvIII or WT EGFR. The exact mechanism of EGFRvIII activation whether mainly through covalent homodimers, heterodimers with WT EGFR, or potential cis-autophosphorylation is still debated (3). It is logical to consider that the failure to attenuate signaling due to inefficient receptor downregulation may be a mechanism by which EGFRvIII is oncogenic as it has impaired degradation, slower internalization, and is recycled efficiently. Studies show that the fully humanized monoclonal antibody panitumumab neutralizes both WT EGFR and EGFRvIII, unlike cetuximab (9, 35). Panitumumab has high binding affinity of ~0.05 nM (avidity effect) that causes bivalent crosslinking, forming stable inactive complexes which induce receptor recycling (re-adaptation) from endosomes to plasma membrane. It also crosslinks EGFRvIII into inactive dimers/oligomers (35). The validated role of this oncoprotein in GBM warrants addressing its biologically consequential and therapeutically tractable role in HNSCC.

**Table 1:** Molecular features: EGFRvIII vs WT EGFR.

Category	WT EGFR	EGFRvIII
Structural Features	Full-length receptor with intact extracellular ligand-binding domain	In-frame deletion of exons 2–7; unique glycine codon at exon 1–8 junction; lacks ligand-binding domain
Tumor Specificity	Expressed in both normal and malignant tissues	Tumor restricted expression
Dimerization Behavior	Ligand-dependent dimerization	Ligand-independent homodimers and heterodimers via free extracellular cysteines
Ligand Binding	Requires EGF (or other ligands) for activation	Cannot bind EGF (lacks binding domain)
Activation	Ligand binding → dimerization → phosphorylation → signaling	Constitutively (“always”) active, ligand-independent
Kinase Activity	High when activated	Lower than ligand-activated WT EGFR
Signaling Pathways Activated	RAS–RAF–MEK–MAPK; PI3K–AKT; JAK–STAT	Constitutive PI3K–AKT and STAT3; gp130 activation via IL-6/LIF secretion
Signaling Output	Strong but transient	Weak but persistent
Internalization	Rapid internalization and lysosomal degradation	Impaired internalization; prolonged surface retention; efficient recycling
Degradation	Ubiquitinated and degraded via Cbl protein	Impaired degradation; remains longer on membrane
Stability on Membrane	Moderate; undergoes degradation after activation	Highly stable; resists degradation

## 3 Reported EGFRvIII frequencies

Multiple studies have surveyed the incidence of the EGFRvIII deletion mutant in HNSCC using diverse methods. **Table 2** summarizes 19 peer-reviewed reports of EGFRvIII expression in HNSCC across years, noting sample size, anatomic subsites, and the detection techniques (IHC, RT-PCR, NGS, etc.) used. It also lists the reported frequency of EGFRvIII and any clinical associations. For example, Chau et al. used quantitative RT-PCR and reported EGFRvIII in ~42% of recurrent/metastatic HNSCC samples, whereas McIntyre et al. developed a hydrolysis-probe qRT-PCR assay validated in GBM to rigorously assess treatment in naïve, site homogenous OSCC and found EGFRvIII in only 2% (25, 39). In contrast, larger studies (e.g. Koch et al., 2017) using multiple primer RT-PCR and sequencing found EGFRvIII to be very rare (<1%) (7). Notably, Melchers et al. (2014) optimized L8A4-antibody IHC and RT-PCR on 531 HNSCCs and detected 0% EGFRvIII (19). The following sections further discuss these vastly differing results regarding EGFRvIII expression and their implications for HNSCC.

**Table 2:** Summary of EGFRvIII detection in HNSCC.

No	Study	Year	No of patient	Country	Subsite	Material	Detection method	Antibody/Primer	Frequency (%)	Positive threshold	Clinical correlation
1.	Jungbluth (36)	2003	10	USA	Metastatic HNSCC	Fresh frozen	IHC	DH8.3	0/10 (0)	Focal: <5% +: >5-25% ++: >25-50% +++: >50-75% ++++: >75%	DH8.3 reactivity was restricted exclusively to glioblastomas
2.	Sok et al. (12)	2006	33	USA	Mixed HNSCC	RT-PCR: Fresh frozen; IHC: FFPE	IHC + RT-PCR	IHC: L8A4 EGFRvIII-specific antibody + specific primers	42% (14/33)	IHC: Strong staining; RT-PCR: Specific amplification	Resistance to cetuximab; increased proliferation
3.	Eriksen et al. (37)	2009	681	Denmark	Locally advanced SCCHN	FFPE	IHC	EGFRvIII antibody	40% (267/675)	Strong immunostaining	Associated with increased tumor size
4.	Yang et al. (24)	2009	39	China	Larynx	Fresh frozen	qRT-PCR (TaqMan)	EGFR and EGFRvIII TaqMan probes F: 5′-GGGCTCTGGAGGAAAAGAAAGG-3′ R: 5′-TCCGTTACACACTTTGCGGC-3′ Probe: 5′FAM-CGACAGCTATGAGATGGAGGAAGACGGCGT-TAMRA3′	15% (6/39)	Relative EGFRvIII mRNA ≥0.025	More frequent in high-EGFR, poorly differentiated
5.	Hama et al. (38)	2009	82	Japan	Various HNSCC sites (oral cavity 26.8%, other sites not specified	Fresh frozen	Sequencing of full-length RT-PCR product	Not specified (full-length EGFR sequencing including extracellular domain)	0% (0/82)	No EGFRvIII variants detected by RT-PCR	Not applicable
6.	Keller (27)	2010	60	USA	Oropharynx (68.3%), Larynx (31.7%)	Fresh frozen	RT-PCR + DNA sequencing	Set 1: 5’-GGCGAGTCGGGCTCTGGAGGAAAAG-3’ and 5’-GGCCCTTCGCACTTCTTACACTTG-3’ (exons 1-8)	23%	In-frame deletion confirmed by sequencing	Significant association with advanced tumor and nodal stage when combined with phosphorylated EGFR
7.	Chau et al. (39)	2011	53	Canada	Mixed R/M HNSCC	FFPE	Quantitative RT-PCR	Exon 4 vs Exon 9 ratio Primers not mentioned	42% (22/53)	Fold change ≥7 vs normal control	Better disease control. but no difference in outcome between erlotinib-treated vs non-erlotinib groups; EGFRvIII not associated with TTP or OS in this cohort.
8.	Tinhofer et al. (8)	2011	47	Germany	R/M HNSCC	FFPE	IHC: L8A4 + RT-PCR validation	IHC: EGFRvIII-specific antibody L8A4 (recognizes junction of exons 1–8 fusion) RT-PCR validation: Yoshimoto protocol for EGFRvIII transcripts	80% (37/46) (any positive expression), 17% (high expression - IHC score ≥7)	IHC score ≥7 for “high” expression (semiquantitative scoring: intensity × extent, max score 12) RT-PCR: 80% concordance with IHC results. RT-PCR confirmed 3/4 IHC + cases, also 1/4 of IHC− cases was RT+ Primers according to Yoshimoto/Mellinghof: 5′-CTTCGGGGAGCAGCGATGCGAC-3′ 5′-ACCAATACCTATTCCGTTACAC-3′ These primers generate a 1044-bp PCR product for the wild-type EGFR transcript, compared with a 243-bp PCR product for the EGFRvIII transcript	High EGFRvIII expression associated with reduced disease control rate (DCR): 13% vs 65% in low expression group (p=0.02) Significantly shorter progression-free survival (PFS): HR 3.3, mean PFS 2.0 vs 5.4 months (p=0.005) No significant association with overall survival Independent predictor of reduced treatment efficacy with cetuximab-docetaxel therapy Multivariate analysis confirmed EGFRvIII as independent predictor for PFS but not OS
9.	Szabo et al. (40)	2011	71	Hungary	Hypopharynx (19), Base of tongue (6), Tonsils (10), Glottis (20), Supraglottic (16)	FFPE	IHC	Mouse monoclonal antibody L8A4 (recognizes junction of exons 1–8 fusion)	21% (15/71)	Positive if cell membrane of tumor cells was stained	Associated with increased EGFR gene copy number (p<0.0001); independent prognostic factor for poor outcome (p=0.041); most mutations affected laryngeal region (8/15)
10.	Smilek et al. (41)	2012	29	Czech Republic	Oral cavity (6.9%), Oropharynx (65.5%), Hypopharynx (17.3%), Larynx (10.3%)	Fresh frozen	RT-PCR	EGFRvIII-fw 5’-AGTCGGGCTCTGGAGGAA3’, EGFRvIII-rev 5’-GCCGTCTTCCTCCATCTCATA-3’, EGFRvIII-probe 5’-ATCACGGCTCGTGCGTCCG-3’ BHQ1-HEX (102 bp)	20.7% (6/29)	Not specified	No significant association with treatment response to cetuximab + radiotherapy; not found in women; no correlation with clinical characteristics except gender
11.	McIntyre et al. (25)	2012	50	Canada	OSCC	FFPE	Real-time PCR (hydrolysis probe) + fluorescent IHC	Novel hydrolysis probe RT-PCR (AQUA^®^ IHC for EGFR)	2% (1/50)	Specific amplification; EGFRvIII only in high-EGFR cases. F: 5′-TCTGCCCGGCGAGTC-3′ R: 5′-GCCGTGATCTGTCACCACATAATT-3′ FAM-labeled probe: 5′-TTTCTTTTCCTCCAGAGCCC-3′	None reported (1 EGFRvIII case in very high EGFR expressors), EGFRvIII-directed therapies are not suitable as first-line options, but may hold value in recurrent or therapy-resistant disease
12.	Wheeler SE et al. (32)	2012	human 52 HNSCC tumor specimens	USA	Oral cavity, oropharynx, hypopharynx, larynx	human tumor tissues, HNSCC cell lines (Cal33, UMSCC1, FaDu) transfected with EGFRvIII; ~8 xenograft mice per group;	RT-PCR and sanger sequencing for EGFRvIII, immunoprecipitation and Western blot for Lyn activation (pY416), siRNA inhibition, xenograft tumor volume	EGFRvIII-specific primers; anti-EGFRvIII antibody 4–5H; Lyn, p-Src (Y416), Fyn, Yes antibodies (Cell Signaling)	12/52 (23%)	Detection of EGFRvIII transcript by RT-PCR (gel band confirmed by sequencing)	EGFRvIII expression correlates with increased Lyn activation, tumor motility, and growth. Dasatinib (Src-family kinase inhibitor) significantly reduced proliferation, migration, invasion, and xenograft tumor volume in EGFRvIII-positive HNSCC. Lyn identified as the primary activated SFK in EGFRvIII+ tumors. Suggests clinical potential of SFK inhibition (e.g., dasatinib) for EGFRvIII+ and cetuximab-resistant HNSCC.
13.	Chang (21)	2013	108	Taiwan	Oral cavity 32.4% tongue, 43.5% buccal mucosa, remaining unspecified oral sites.	FFPE	IHC	WT EGFR (Novocastra RTU-EGFR-384), EGFRvIII (4-5H), PTEN (138G8), pAKT (Ser473 D9E)	75% (31.5% high expression)	>5% membranous (WT EGFR), >5% cytoplasmic (EGFRvIII, PTEN, pAKT); EGFRvIII scored 0–4+ Classified into five groups according to the extent of moderate to strong cytoplasmic reactivity: 0, (none); 1+, (1 ~ 24%); 2+, (25 ~ 49%); 3+, (50 ~ 74%); and 4+, (75 ~ 100%)	EGFRvIII correlated with tumor T classification and TNM stage; EGFRvIII and pAKT were independent predictors of patient survival
14.	Boeckx et al (42)	2014	52	Belgium	Oropharynx (27 patients), Larynx (25 patients)	FFPE	One-step real-time RT-PCR	EGFRe1-F: 5’-GAGTCGGGCTCTGGAGGAA-3’, EGFRe8-R: 5’-GGCCCTTCGCACTTCTTACA-3’, EGFRe1/8-TM probe: 6-FAM-AAAGGTAATTATGTGGTGACAGATCACGGCTC-BHQ-1	0% (0/44 evaluable samples)	Not specified	No EGFRvIII mutations detected; study concludes EGFRvIII unlikely to play major role in predicting anti-EGFR therapy response in Belgian HNSCC population
15.	Melchers et al. (19)	2014	531	Netherlands	Oral cavity 272 (52%) Oropharynx 205 (39%) Hypopharynx 8 (2%) Larynx 37 (7%)	FFPE	IHC (L8A4) + RT-PCR	EGFRvIII antibody L8A4; EGFRvIII RT-PCR primers	0% (0/531)	– (no true positives)	EGFRvIII absent – no impact on prognosis
16.	Khattri et al. (20)	2015	638	USA	Mixed HNSCC	FFPE; OCT frozen	qRT-PCR (108 samples), RNA-seq (432 samples), IHC (105 samples)	Multiple EGFRvIII breakpoint PCR sets (L8A4 IHC)	0.31% (2/638)	Varies by assay	Very rare – clinical significance unclear
17.	Wheeler et al. (26)	2015	25 (subset of a 154-case TMA cohort)	USA	Various HNSCC and glioma tissues	Fresh frozen FFPE	RT-PCR, IHC, Exon junction sequencing, Long-range PCR, RNAseq	EGFRvIII-specific primers; IHC antibodies	9/25 (36%)	Confirmed by sequencing (exon 1–8 joining)	EGFRvIII expression independent of EGFR amplification; low expression; potential negative predictive biomarker for cetuximab, EGFRvIII detection depends strongly on assay design and tissue coverage and that RNAseq/automated fusion callers may miss EGFRvIII.
18.	TCGA Comprehensive genomic characterization of HNSCC (43)	2015	279	International	Mixed HNSCC	Fresh frozen	Whole-genome + RNA sequencing	Hybrid capture exome libraries and random priming	0.4% (1/279	q < 0.1 used for significantly mutated genes	Rare structural evidence of EGFRvIII by WGS/RNA-Seq analyses
19.	Koch et al. (7)	2017	149	Germany	Mixed HNSCC (Oral cavity 13.4%, Oropharynx 36.2%, Hypopharynx 8.7%, Larynx 17.4%, Sinonasal 24.2%)	RT-PCR: Fresh frozen; IHC: FFPE	RT-PCR + Sequencing + IHC	Five EGFRvIII primer sets in parallel: Primer 1: 5′-GGCTCTGGAGGAAAAGAAAG-3′/5′-TCCTCCATCTCATAGCTGTCG-3′ Primer 2: 5′-TGCTGGCTGCGCTCTGC-3′/5′-CACAGGCTCGGACGCAC-3′ Primer 3: 5′-GGAGCAGCGATGCGACCCTC-3′/5′-ACACTTGCGGACGCCGTCTT-3′ Primer 4: 5′-ATGCGACCCTCCGGGACG-3′/5′-ATTCCGTTACACACTTTGCGGC-3′ Primer 5: 5′-GAGCTCTTCGGGGAGCAG-3′/5′-GTGATCTGTCACCACATAATTACCTTTCT-3′ L8A4 EGFRvIII-specific antibody for IHC	~0.7% (1/149)	PCR: Multi-primer confirmation required; IHC: Strong staining; Sequencing: Confirmation of exon 2–7 deletion	No significant difference in overall survival between EGFRvIII-positive and negative cases (p = 0.618). Study concluded EGFRvIII is not relevant for targeted therapy and radiation in HNSCC. *In vitro* experiments showed similar response rates between EGFRvIII-transfected and wild-type cells to EGFR inhibitors and radiation.
20.	Peng et al. (22)	2025	7	Taiwan	OSCC specimen samples, cell lines	FFPE, OSCC cell cultures; mice xenografts	RT-PCR, Sanger sequencing, Western blot, qPCR	EGFRvIII-specific primers/antibody	70% (5/7)	Presence of 128-bp PCR product or protein band	EGFRvIII expression associated with tumor aggressiveness, fibroblast activation, increased migration/invasion

## 4 Sources of heterogeneity in EGFRvIII literature

### 4.1 Detection strategies and technical pitfalls

The dramatic variation in reported EGFRvIII frequencies across HNSCC studies stems from multiple interconnected sources of heterogeneity that confound direct comparisons and clinical interpretation. Technical variability represents a primary source of discordance. Different detection techniques have been used in the past for EGFRvIII, such as western blot analysis, immunohistochemistry (IHC), immunofluorescence (IF), enzyme-linked immunosorbent assay (ELISA), and flow cytometry analysis (FACS). Among these, IHC and RT-PCR (conventional or qPCR) were most common (7, 8, 12, 19, 20, 25, 26, 39). Real-time PCR appears to be a very specific and sensitive method of EGFRvIII detection, while IHC analyses returned a number of false positives when using L8A4 antibody (44) highlighting how detection methodology fundamentally impacts reported frequencies and emphasizing the critical need for standardized protocols and antibody validation. Although EGFRvIII mutation has been detected at the mRNA level using quantitative real-time PCR in several studies, no significant association with treatment response to anti-EGFR therapy was found (41). This may reflect the limitation that mRNA expression is not a reliable proxy for the presence or functionality of therapeutically relevant EGFRvIII protein. No HNSCC-specific ddPCR or NGS-panel study has reported high EGFRvIII rates, and a recent large NGS survey found EGFRvIII almost exclusively in GBMs and not in head and neck tumors (11). Koch’s study screened 149 HNSCC specimens for EGFRvIII using RT-PCR with five different primers, sequencing, and immunohistochemical staining. The primers produced highly inconsistent results likely from nonspecific off-target amplification and tissue age effects, and only one sample was positive by all methods. Using a less stringent criterion (positivity by any method), EGFRvIII was detected in 13 of 149 cases, with rates of 4.4% in older formalin-fixed paraffin-embedded (FFPE), 19.2% in recent FFPE, and 30% in fresh-frozen specimens (7). The studies above illustrate the technical challenges (antibody specificity, primer design, and tissue quality) and the resulting frequency discrepancies (7, 39). Koch et al. and Khattri et al. reinforced that EGFRvIII is extremely rare in HNSCC when using multiple primers or sequencing (7, 20). The Chau et al. study used a quantitative RT-PCR approach measuring exon ratios, which is more standardized than earlier methods (39). Using one-step real-time PCR on FFPE material, no EGFRvIII mutations could be detected (42). The probe-based real-time PCR detection of EGFRvIII was found in approximately 70% of OSCC (25). Wheeler et al. found that accurate detection requires multiplex PCR with specific primers, Sanger sequencing confirmation, and manual review of RNAseq when applicable (26). Emerging assays like targeted hybrid-capture NGS and digital PCR can in principle detect the exon 2–7 fusion, but so far, have confirmed that EGFRvIII is infrequent in HNSCC compared to GBM (45, 46).

**Figure 2:**
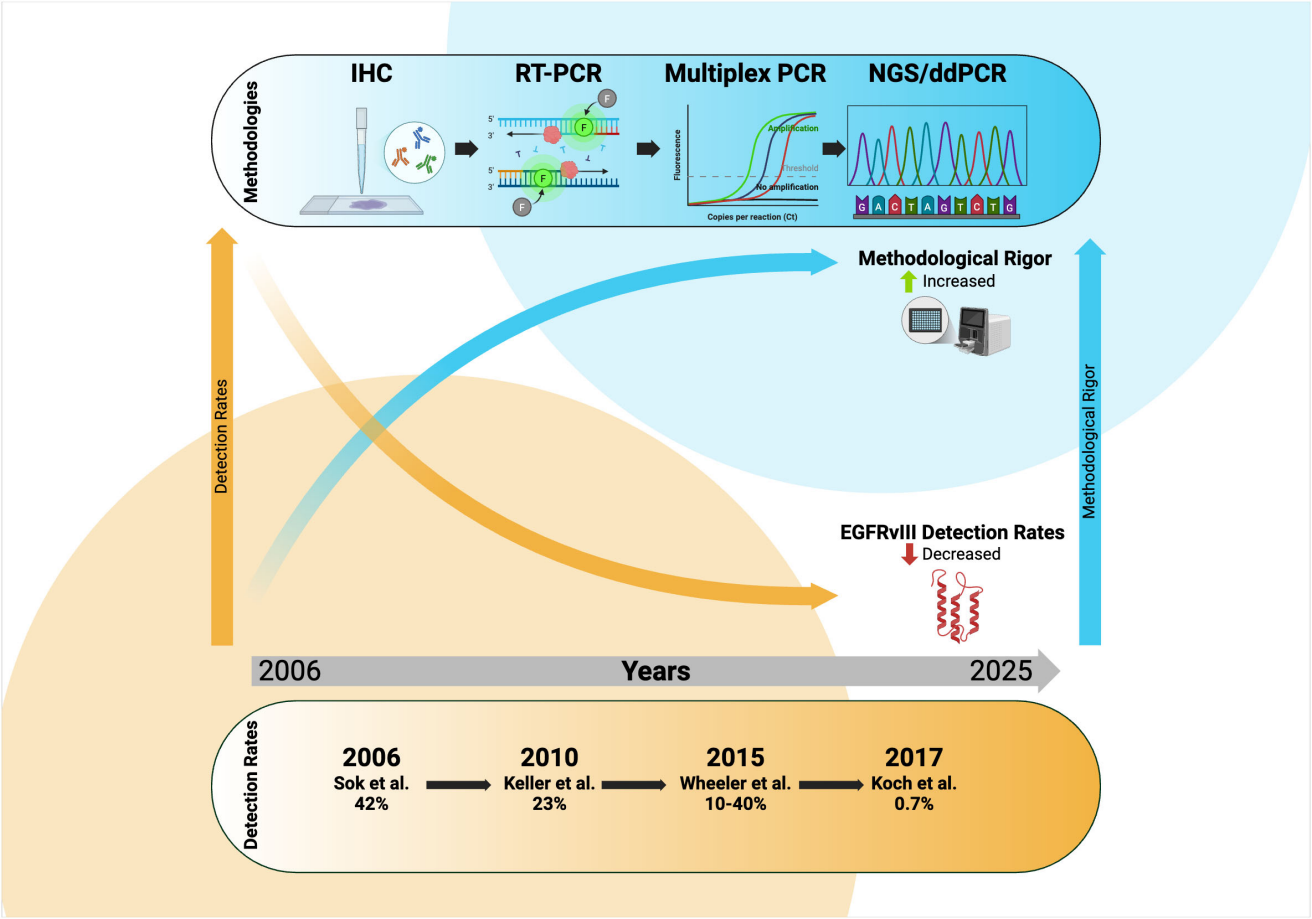
Temporal relationship between EGFRvIII detection rates and methodological rigor in HNSCC studies.

The temporal evolution of EGFRvIII detection methodologies in HNSCC reveals a striking inverse correlation between methodological rigor and reported frequency, fundamentally challenging the clinical significance of this variant in head and neck malignancies (**Figure 2**). Early studies (2006-2011) predominantly employed single detection techniques with commercially available but inadequately validated antibodies, consistently reporting high EGFRvIII frequencies ranging from 17% to 42% (Sok et al. 42%, Eriksen et al. 34%, Chau et al. 42%) (12, 37, 39). These pioneering investigations, while groundbreaking in identifying potential EGFRvIII expression in HNSCC, were methodologically limited by reliance on single-platform detection approaches with the use of antibodies that subsequent validation studies have demonstrated to produce significant false-positive results. In stark contrast, later studies (2012-2025) implemented increasingly rigorous methodological approaches incorporating multiple orthogonal detection platforms, stringent validation protocols, and enhanced primer specificity, resulting in dramatically reduced reported frequencies ranging from 2% to complete absence (Wheeler et al. 0.31%, Melchers et al. 0%, Boeckx et al. 0%) (19, 26, 42). This temporal shift in methodology coincided with improved understanding of EGFRvIII biology, enhanced antibody specificity testing, and the recognition that authentic EGFRvIII detection requires multiple confirmatory approaches to distinguish true variants from technical artifacts.

When looking at each detection method’s variability, real-time PCR methodologies demonstrated significant variability depending on primer design, amplification conditions, and detection chemistry. Simple RT-PCR approaches using basic primer sets (as employed in early studies) lack the specificity and sensitivity required for reliable EGFRvIII detection. While quantitative real-time PCR with validated TaqMan probes (24) or hydrolysis probe systems (25) provides enhanced specificity, it requires careful optimization for the unique amplification characteristics of EGFRvIII transcripts. Different primer sets spanning the deletion junction produce discordant results due to differences in amplicon size, melting temperature, and specificity (20). No consensus primer set has been validated across multiple independent laboratories for HNSCC (7), in contrast to GBM where standardized primer sequences are widely adopted. Yang et al’s study (24) represents a technically refined approach to EGFRvIII detection by using a validated real-time qPCR assay with mutation-specific probes and a recombinant positive control, thereby improving reliability over earlier immunohistochemical or conventional PCR-based reports. However, the absence of protein-level verification, RNA instability, and tumor heterogeneity remain key pitfalls that may explain the variable and often low detection rate of EGFRvIII in HNSCC. Low and unstable EGFRvIII mRNA in archival or frozen samples, unreported RNA integrity, and signal dilution from homogenized RNA samples likely contribute to underestimation of prevalence. Given high cycle numbers (35 cycles) (24) and construction of plasmid controls (key quality control step), there’s potential for carryover contamination if strict PCR hygiene isn’t maintained. The critical technical challenge lies in primer design that specifically amplifies the exon 2–7 deletion junction without cross-reactivity to WT EGFR or other EGFR variants, necessitating multiple primer set validation and orthogonal confirmation.

IHC detection of EGFRvIII faces substantial technical obstacles, primarily centered on antibody specificity and availability. The widely used L8A4 antibody has demonstrated significant cross-reactivity issues leading to false-positive results (9), with many EGFRvIII-specific antibodies utilized in early studies no longer commercially available, preventing reproducibility and validation of historical findings. Even though several monoclonal antibodies specific for EGFRvIII have been described, their availability is limited by licensing and patent issues (47). The commonly used antibodies like DH8.3, 528, and 806 showed high off-target binding, were not tumor-specific, and some were limited to brain tumors (DH8.3) (36). Comparatively, mAb 806 recognizes conformationally exposed EGFR epitope in amplified/overexpressed/mutant EGFR, which are strongly positive in SCCs of head, neck, lung, esophagus, and bladder (36). McIntyre coupled RT-PCR with quantitative AQUA^®^ IHC, that establishes a gold-standard, automated, observer independent framework for accurate EGFR/EGFRvIII assessment that yields highly reproducible protein expression data on a continuous scale in clinical diagnostics over conventional semi quantitative IHC (25). Furthermore, IHC interpretation requires careful distinction between membrane staining intensity and non-specific cytoplasmic reactivity, with inconsistent scoring systems and intra/inter observer variability across studies contributing to frequency discrepancies. Even though IHC is a very common protein based method for spatial information and direct protein detection and validation, the use of only a single method for EGFRvIII detection and failure in the technical implementation are met with criticism. This explains why more recent studies have concluded that EGFRvIII is extremely rare in HNSCC and the clinical significance remains unclear. Another important root cause for detection controversy is the absence of validation standards. Unlike p16 for HPV status (48) or PD-L1 scoring (49), there are no equivalent consensus guidelines that exist for EGFRvIII detection in HNSCC.

EGFRvIII detected by RT-PCR does not always show corresponding protein expression by IHC (8, 12). Conversely, the positive EGFRvIII positive Immunohistochemical expression without detection of the mRNA transcript can also occur (9). Because of this inconsistency, the most methodologically robust studies employed combined detection approaches utilizing both IHC and RT-PCR platforms to provide orthogonal validation of EGFRvIII expression (9). Therefore, integrating transcript and protein assays remains essential for definitive EGFRvIII characterization in HNC. TCGA HNSCC comprehensively profiled somatic mutations, structural alterations etc. in 279 tumors, and the low prevalence of EGFRvIII reported (1/279) reflects the sensitivity and broad scope of unbiased whole genome and transcriptome approaches (43). These multi-platform studies consistently report the lowest frequencies, suggesting that single-platform approaches may overestimate true EGFRvIII prevalence due to technical artifacts. RNA sequencing and targeted sequencing approaches represent the most definitive detection methods, providing sequence-level confirmation of the specific exon 2–7 deletion, but remain limited in clinical application due to cost and technical complexity. Use of positive and negative controls with objective quantification based on cycle threshold (Ct) values in real time qPCR, reduces subjective interpretation and inter observer errors inherent to IHC. The detection of EGFRvIII demonstrates significant sensitivity to sample preparation and preservation methods, with FFPE tissues showing reduced detection sensitivity compared to fresh-frozen samples due to RNA degradation and cross-linking artifacts. This preservation dependent detection variability may contribute to frequency discrepancies between studies utilizing different tissue processing protocols, particularly when considering that EGFRvIII often requires concurrent EGFR gene amplification to achieve detectable expression levels which makes the variant particularly susceptible to sample quality-related detection failures. FFPE yields dramatically different RNA quality due to fragmentation, chemical modification, and reduced amplifiable template (50). This issue with archival blocks of uncertain age and storage conditions in many of the retrospective HNSCC studies underscores an important need for standard operating procedures (SOPs) across labs which could otherwise lead to uncontrolled pre-analytical variables. **Table 2** presents the diagnostic techniques addressed in each article.

### 4.2 Anatomical subsite and etiological and tumor biological features of EGFRvIII expression in HNSCC:

**Figure 3:**
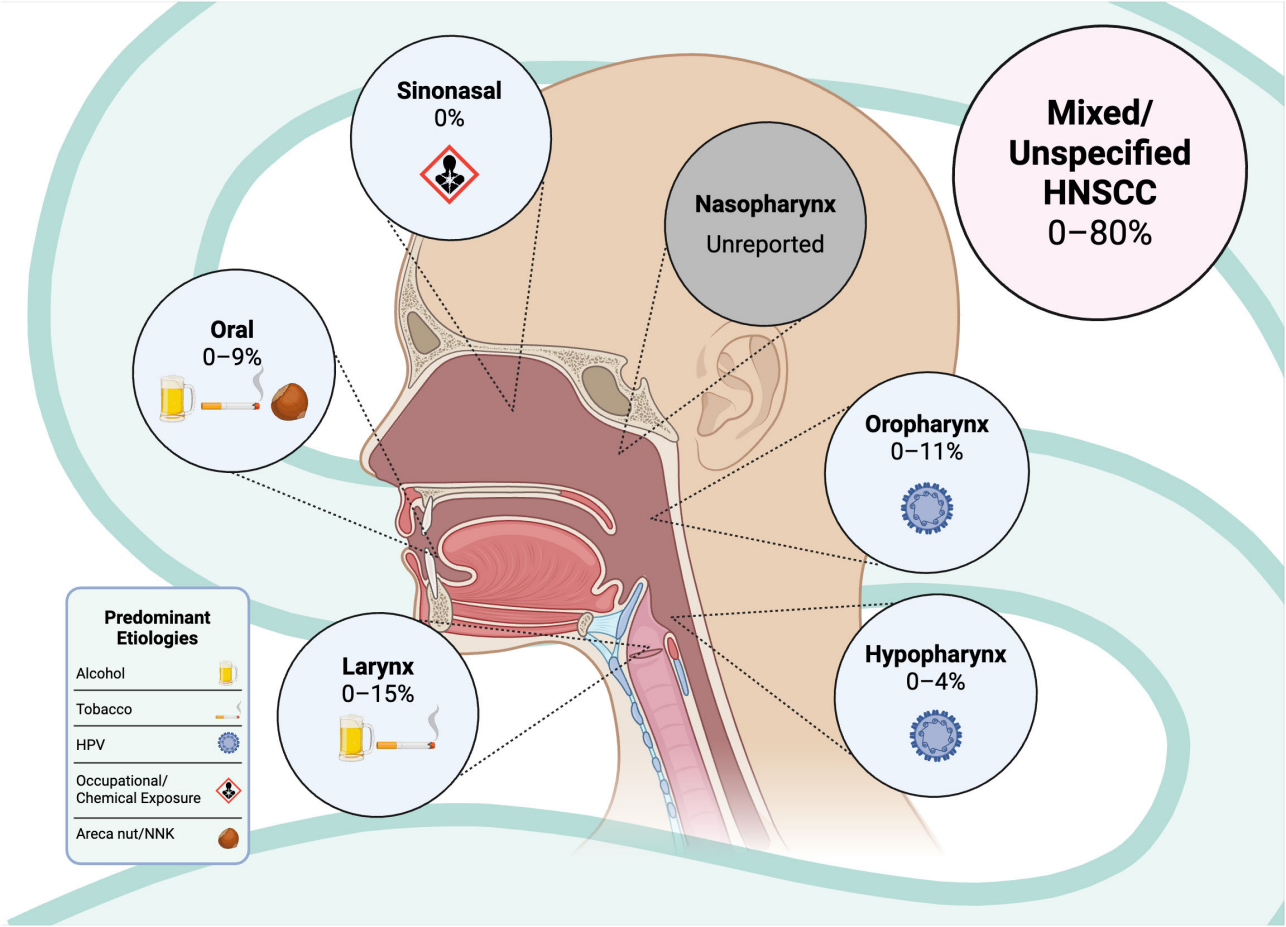
Site-specific distribution of reported EGFRvIII detection rates across HNSCC.

The level of EGFR protein expression in HNSCC varies considerably across anatomical subsites. For example, carcinomas of the pharynx and oral cavity generally exhibit higher EGFR expression compared with those arising from the larynx (51). Given this subsite-dependent variability in EGFR expression, it is plausible that the frequency of EGFRvIII also differs among distinct HNSCC subsites (**Figure 3**). However, despite extensive investigation of EGFR alterations in HNSCC, there remains no consensus on the prevalence of EGFRvIII expression (52). To date, only very few studies have systematically evaluated EGFRvIII frequency across specific subsites, including malignancies of the oral cavity, pharynx, and larynx. Considering that the head and neck region comprises several biologically distinct mucosal sites such as the oral cavity, pharynx, paranasal sinuses, and larynx, the limited availability of subsite-stratified data represents a critical gap in the literature. This marked intraregional heterogeneity within HNSCC may partially account for the conflicting EGFRvIII frequency data reported across different studies. Melchers et al. have assessed the prevalence of EGFRvIII in three well defined cohorts of HNSCC of OSCC, oropharyngeal squamous cell carcinoma (OPSCC), and various other locations which are a POSTOP cohort (19) and concluded EGFRvIII is not usable as a prognostic marker due to its lack of presence.

Oral cavity and larynx cancers are generally associated with tobacco consumption, alcohol abuse or both, whereas oropharynx cancers are increasingly attributed to infection with human papillomavirus (HPV), primarily HPV-16 (53). This etiological distinction is crucial for EGFRvIII analysis, as the molecular landscape differs significantly between HPV-positive and HPV-negative tumors. HPV-driven carcinogenesis fundamentally alters the molecular landscape of OPSCC, which may be relevant to EGFRvIII analysis of OPSCC. The 8th edition UICC/AJCC staging system (54) now separates HPV+ OPSCC from HPV-negative disease, formally recognizing these as distinct clinical entities that likely require separate molecular and clinical characterization as it is one of the most rapidly increasing incidences of any cancer in high-income countries (55).

Peng et al. (22) showed that environmental carcinogen interaction like NNK (nicotine-derived nitrosamine ketone) and arecoline increase EGFRvIII expression and downstream oncogenic signaling, enhancing tumor aggressiveness. The influence of regional carcinogen exposure patterns, including tobacco use profiles, areca nut chewing, alcohol consumption patterns, HPV prevalence, and occupational or environmental exposures, may further contribute to geographic clustering of EGFRvIII frequencies through differential selective pressure on EGFR pathway alterations during carcinogenesis (56). Chang et al. (21) investigated OSCC in a betel nut prevalent population, demonstrating how etiological exposures contribute to regional heterogeneity in tumor biology. Their study showed that EGFRvIII drives tumor cell proliferation while concurrently remodeling the tumor microenvironment through secretion of lipocalin-2 (LCN2), which activates surrounding fibroblasts via STAT3 signaling to establish a tumor-supportive niche. Notably, environmental carcinogens such as nicotine derivatives and arecoline were found to upregulate EGFRvIII expression, underscoring the therapeutic potential of targeting EGFRvIII and its downstream pathways in aggressive OSCC (22).

The anatomical subsite distribution of EGFRvIII expression in HNSCC reveals significant heterogeneity–yet robust data are largely lacking in existing studies. Within available studies, 15.4% (6/39) of laryngeal carcinoma cases demonstrated EGFRvIII mRNA expression that correlated with EGFR overexpression and poor differentiation (24), while OSCC showed markedly higher prevalence with approximately 70% of tumor samples demonstrating high EGFRvIII expression (22, 57). This striking subsite-specific variation, combined with the distinct molecular profiles of HPV-positive versus HPV-negative oropharyngeal cancers along with the different etiological backgrounds across oral cavity, laryngeal, and hypopharyngeal tumors, underscores the critical need for anatomical and etiological stratification in EGFRvIII analysis to avoid obscuring important biological relationships when pooling heterogeneous HNSCC data (58). Thus the scarcity of subsite-specific EGFRvIII data represents a critical knowledge gap. EGFR is overexpressed in up to 90% of HNC (59), yet EGFRvIII expression patterns across these anatomically and etiologically distinct tumor types remain poorly characterized. It is also observed that nasopharyngeal carcinoma and other less common HNSCC subsites are completely absent from EGFRvIII literature. Considering the distinct etiologies of these tumors (EBV association in nasopharyngeal cancer, occupational exposures in sinonasal cancers) relative to other HNC subsites highlights an urgent need for systematic subsite-stratified analysis of EGFRvIII expression in future research endeavors.

Tumor biology introduces additional complexity through significant intra-tumor heterogeneity and clonal evolution (60). GBM exhibits subpopulations with heterogeneous EGFRvIII expression that cooperate to drive tumor progression and treatment resistance (61), a phenomenon likely extending to HNSCC. Despite the conflicting opinions regarding EGFRvIII’s biological significance ranging from negligible functional importance to a marker of advanced malignancy, preclinical data shows that EGFRvIII expressing cells promote proliferation, invasion, angiogenesis, stemness, and therapy resistance – many of the hallmarks of cancer – suggesting EGFRvIII may represent a late evolutionary event whose prevalence varies with disease progression and stage (3, 10). Consequently, single-site or single-time point sampling may inadequately capture the true EGFRvIII status of heterogeneous tumors, potentially missing EGFRvIII-positive clones and underestimating their role in disease progression and treatment failure.

### 4.3 Geographical and population basis for variation

**Figure 4:**
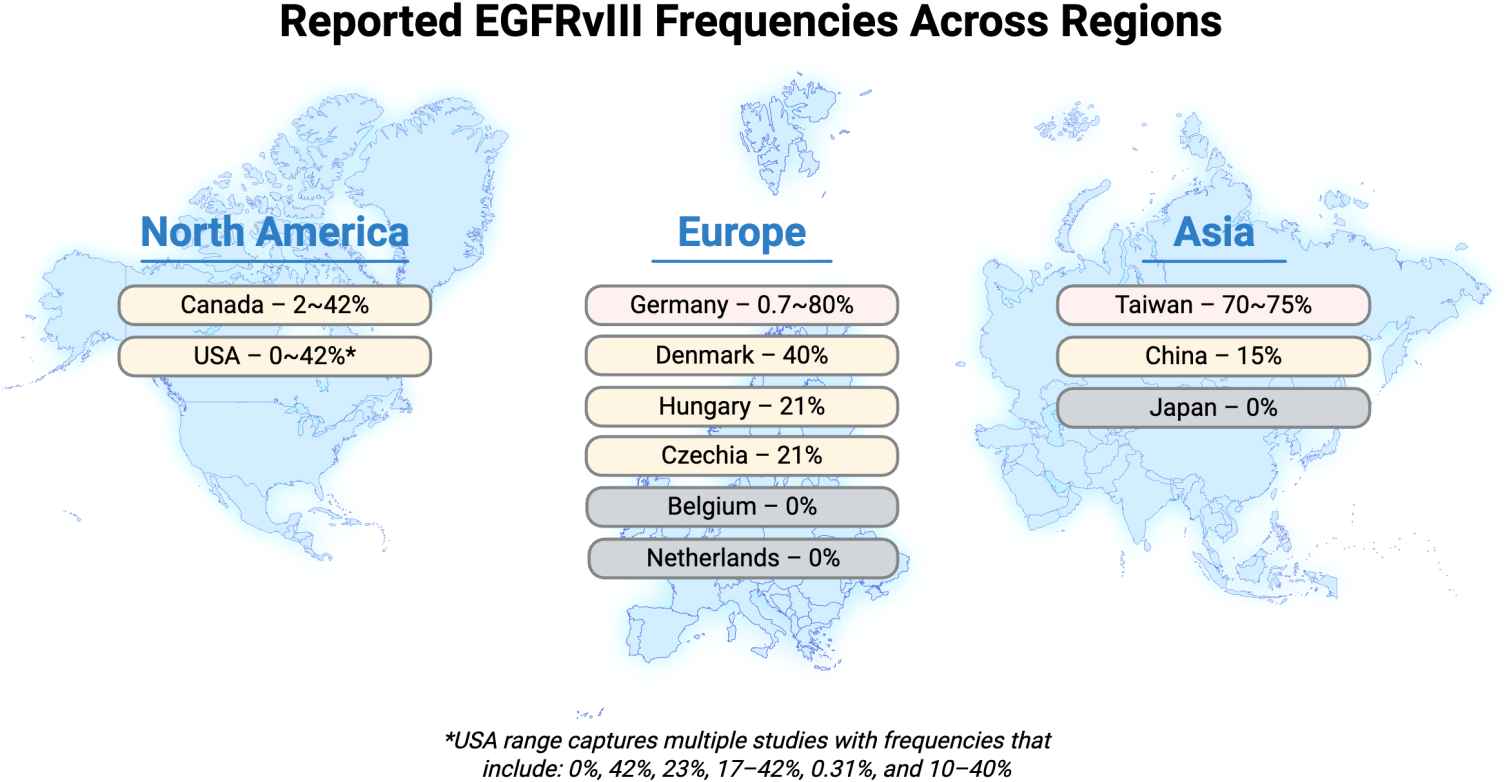
Reported EGFRvIII frequencies across geographic regions.

Geographic variations may reflect different populations and methodologies of EGFRvIII analysis. Hama et al. (38) focused on full-length EGFR sequencing rather than specific EGFRvIII detection methods and notes the contrast with previous reports of EGFRvIII mutation rate in HNSCC, suggesting most EGFR mutations in Japanese HNSCC patients lie within kinase domains (E709K, V765G, Ins770G, and G1022S) rather than the extracellular domain (38). Population and environmental factors add additional variability, as the frequency of EGFRvIII in specific subsites of HNSCC is disputed and may vary across different geographic or ethnic populations (56, 58). The data presented in **Table 2** and **Figure 4** demonstrates striking geographic clustering where Asian studies predominantly report either very high frequencies (70%-75%) or complete absence (0%), while European (Germany, Denmark, Hungary, Czechia), studies show intermediate frequencies (20-40%) and North American studies display a broad range (0-42%), indicating that geographic, ethnic, and anatomical variations require systematic stratification in study design. The established pattern of ethnic variation in EGFR mutations, where Asian populations demonstrate markedly different mutation frequencies compared to Caucasian populations (1% of Caucasians versus 7% of Asians with HNSCC EGFR mutations) (58), extends to EGFRvIII variants and may reflect underlying genetic polymorphisms, environmental exposures, or gene-environment interactions that differentially predispose certain populations to specific EGFR alterations. Yang et al. used a validated specific and sensitive real-time quantitative PCR assay with TaqMan probe on 39 Chinese patient samples and confirmed the expression of EGFRvIII in a few laryngeal carcinoma tissues (24).

Notable heterogeneity has been reported within European populations, as EGFR overexpression was frequently and consistently noted in Austrian, Spanish, and Dutch cohorts, while it was infrequent in Swedish, French, and Italian populations, suggesting that continental-level genetic stratification may be insufficient and that more granular population-specific analyses are necessary to understand the true distribution of EGFRvIII variants (58).

Finally, study design limitations significantly contribute to heterogeneity through retrospective designs with inherent selection biases, variable sample sizes that may inadequately power subgroup analyses, inconsistent cohort composition regarding disease stage and treatment history, and lack of standardized outcome definitions, collectively creating a literature landscape where methodological differences may be as influential as biological factors in determining reported EGFRvIII frequencies and clinical associations. When true prevalence is below 5%, even cohorts of 100–200 patients lack adequate power to detect meaningful clinical associations (20).

## 5 Clinical and prognostic implications

The clinical and prognostic significance of EGFRvIII expression in HNSCC presents a complex landscape of mixed results that reflects both the biological heterogeneity of HNSCC and methodological variations across studies. EGFRvIII expression has been observed in poorly and moderately differentiated tumors rather than in well differentiated tumors, showing its relevance in the aggressiveness of cancer (24). Chang et al. (2013) showed a significant association between EGFRvIII & tumor stage (T) (P<0.001) where high expression is more frequent in stage 3/4 (40.7%) vs stage 1/2 (22.2%) (21). Tinhofer et al. (8) detected high EGFRvIII (IHC score ≥7) expression in 17% of HNSCC cases and used cetuximab-docetaxel therapy to demonstrate significant association with reduced disease control and shorter progression-free survival (PFS), where patients with low EGFRvIII immunohistochemical scores achieved a better disease control rate (DCR) of 65% compared to only 13% in patients with high EGFRvIII scores (p=0.02). This suggests that EGFRvIII expression may serve as a negative predictive biomarker for conventional therapy response in recurrent/metastatic HNSCC. Their findings are among the few studies that found a significant clinical correlation between EGFRvIII expression and treatment outcomes, specifically showing worse response to cetuximab-based therapy in patients with high EGFRvIII expression. Contrastingly, in a study of 149 HNSCC patients (7), patients with EGFRvIII detection showed no significant difference in overall survival compared to those without EGFRvIII detection (p = 0.618), indicating that EGFRvIII expression is not associated with prognosis in HNSCC. This is confirmed by a review done by Bossi et al. (62) in 2016 that concluded EGFRvIII showed no value under prognostic or predictive categories as there was *no correlation* with overalll survival, disease free survival (DFS) or recurrence. Sok et al. detected EGFRvIII in 17–42% of HNSCC cases, which predicted resistance to cetuximab and increased tumor cell proliferation in mixed head and neck cohorts (12). Co-expression of EGFRvIII and phosphorylated EGFR correlated with advanced T and N stage (27), and EGFRvIII immuno-positivity served as an independent poor prognostic factor in laryngeal cancers (40). In OSCC, EGFRvIII associated with higher TNM stage and inferior patient survival (21) which is a finding reinforced by preclinical data linking EGFRvIII to stromal activation and enhanced invasion (22). Although EGFRvIII shows a relatively low positivity rate in laryngeal carcinoma, its expression in Yang et al’s study appears tumor-specific and is more frequently observed in EGFR-overexpressing, poorly differentiated tumors. This suggests a potential association between EGFRvIII and aggressive or less differentiated phenotypes in HNSCC (24, 39). In the study by Smilek et al. *(*41*)*, EGFRvIII was detected in approximately 20% of HNSCC cases, but no association was found between EGFRvIII expression and response to cetuximab combined with radiotherapy. Interestingly, higher EGFR mRNA copy number correlated with complete response, suggesting that elevated EGFR expression rather than EGFRvIII mutation predicts sensitivity to EGFR-targeted therapy. These results contrast with earlier studies linking EGFRvIII to therapeutic resistance, emphasizing that EGFRvIII’s clinical significance may depend on tumor subsite, disease stage, and detection method. Together with evidence from other cohorts, these findings support the notion that EGFRvIII is biologically relevant but not uniformly predictive of treatment outcomes in HNSCC.

An interesting paper from Chau et al. (39) was the first study to evaluate EGFRvIII in recurrent/metastatic (R/M) HNSCC patients treated with or without EGFR tyrosine kinase inhibitors (TKIs) and showed EGFRvIII in 42% of 53 R/M HNSCC along with unexpected association with better disease control regardless of treatment type. This contradicted the hypothesis that EGFRvIII would predict poor outcomes as no association with overall survival or time to progression was observed (39). This is also similar to GBM, where the role of EGFRvIII as a prognostic or predictive biomarker for EGFR inhibitor response remains controversial. While EGFRvIII and PTEN co-expression has been linked to improved response to EGFR TKIs in some glioma cohorts (63), other studies have reported EGFRvIII as a marker of poorer survival (64, 65) or found no prognostic significance (66). Associations with traditional clinicopathological features including tumor size, nodal status, stage, and grade remain inconsistently reported across studies. Nevertheless, EGFRvIII contributes to enhanced tumor growth and resistance to WT EGFR targeting, indicating that the antitumor efficacy of EGFR targeting strategies may be enhanced by the addition of EGFRvIII-specific blockade (18) which has particular relevance for panitumumab treatment outcomes in the recurrent and metastatic setting.

## 6 Therapeutic potential and challenges

In addressing EGFRvIII as a precision‐oncology target in HNSCC, implementing ultrasensitive assays such as digital droplet PCR and liquid biopsy based ctDNA monitoring is essential to capture its low‐abundance expression and clonal dynamics under therapeutic pressure (67). Despite the ability of modern RNA-seq platforms to robustly detect EGFRvIII in glioblastoma, its low abundance, subclonal architecture, and exon-skipping configuration in HNSCC likely constrain reliable detection by conventional bulk RNA-seq pipelines, even at current sequencing depths, thereby necessitating junction-focused or targeted assays for accurate identification (26, 45, 68, 69). While it is beyond the scope of this review to comprehensively assess the therapeutic potential and challenges of EGFRvIII, its clinical utility in other tumors should not be overlooked. Emerging bispecific antibodies (70) and antibody drug conjugates (71) that exploit the shared cryptic epitope on EGFRvIII and overexpressed WT EGFR are currently entering early‐phase HNSCC trials, achieving selective cytotoxicity while sparing normal tissues. Quantitative flow cytometry estimates of receptor density per cell in native tumors measured 2.7–6.8 × 10^5^ EGFRvIII receptors per cell (72), which established that native tumor cells express enough receptor density for effective antibody-based targeting *in vivo*. The identification of antibody 806 – a reagent that recognizes a tumor-specific conformational epitope on amplified or mutant EGFR but spares normal tissues – provides a compelling rationale for selective EGFR-targeted immunotherapy (36). Since EGFRvIII shares similar conformational alterations and membrane exposure patterns, mAb806 and its derivatives may offer a strategy to selectively target EGFRvIII-positive or EGFR-overexpressing HNSCC while minimizing systemic toxicity. Liquid biopsies enable real‐time surveillance of EGFRvIII‐positive subclones and guide rational combination regimens designed to thwart bypass signaling through MET or PDGFR pathways. Armored CAR T cells engineered with dominant‐negative TGFβR or IL-12 expression demonstrate enhanced tumor infiltration and persistence in solid tumor microenvironments (73), though cytokine‐related toxicities and immunogenicity require vigilant monitoring. Finally, neo-antigen vaccines, when combined with immune checkpoint blockade directed against the EGFRvIII junctional peptide, hold promise for overcoming intratumoral heterogeneity and achieving durable remissions (74, 75). Recent preclinical work with the recombinant immunotoxin DT390-BiscFv806 demonstrated picomolar‐level cytotoxicity against EGFRvIII cells while sparing normal EGFR, and its antitumor efficacy increased with greater total EGFR expression, supporting a dual-targeting EGFR/EGFRvIII strategy in HNSCC despite low EGFRvIII prevalence (76). Monoclonal antibody mAb806 binds a cryptic epitope exposed on both EGFRvIII and overexpressed WT EGFR, preferentially recognizing immature high-mannose forms and highlighting structural vulnerabilities from aberrant glycosylation and mislocalization (77, 78). Targeting the EGFRvIII–LCN2–STAT3 axis offers a dual approach to disrupt tumor‐intrinsic signaling and the supportive microenvironment by which OSCC progression can be mitigated with improved therapeutic outcome (22). EGFRvIII expression increased class IVa beta-tubulin mRNA by 2.5-fold and class IVb by 3.1-fold while conferring paclitaxel resistance, suggesting that inhibition of EGFRvIII kinase activity could partially reverse drug resistance and enhance chemotherapy efficacy in EGFRvIII-expressing malignancies (79).

EGFR806-CAR T cells successfully targeted a tumor-restricted EGFR epitope (the mAb806 epitope exposed on untethered EGFR and EGFRvIII) with selectivity for GBM cells while sparing normal tissues expressing WT EGFR, achieving 50-100% survival in orthotopic glioma models without toxicity to EGFR+ normal tissues. CARv3-TEAM-E T cells targeting both EGFRvIII and WT EGFR through a secreted T-cell engaging molecule produced dramatic and rapid tumor regression within days in all three recurrent GBM patients without grade 3+ toxicity, though responses were transient in two of three patients (80, 81). EGFR806-CAR approach would be especially attractive for HNSCC since it could target tumor cells with aberrant EGFR conformations while potentially sparing normal epithelial tissues that express properly tethered WT EGFR, addressing a major toxicity concern in treating epithelial-rich tissues of the head and neck region. Prognostic associations with OS and DFS remain inconsistent, underscoring the need for standardized detection and patient stratification. Mechanistically, EGFRvIII stability or dimerization, not merely kinase activity, alongside cross-activation of HGFR/PDGFR, PI3K/AKT, SRC kinases, and MAPK cascades drives resistance (3, 9, 82), necessitating combination regimens that simultaneously inhibit EGFRvIII and compensatory pathways (83).

## 7 Overarching perspectives & future directions

The EGFRvIII saga in HNSCC embodies the challenges of rare biomarker validation. There was initial optimism fueled by inadequate methodology, subsequent skepticism as technical limitations surfaced, and ongoing uncertainty due to lack of definitive studies. This trajectory is not unique as similar arcs have characterized other rare oncogenic drivers before either validation (NTRK fusions) or abandonment (initial MET exon 14 skipping reports in various cancers prior to rigorous validation in lung cancer) (84–86). From the results above, it is clear that the controversy around EGFRvIII in HNSCC stems from multiple interconnected factors like technical sensitivity and specificity issues, primer set variability, sample type and quality heterogeneity, low prevalence and statistical power, and absence of validation standards. Considering these variables across the entirety of HNSCC literature calls into question the early reports of high EGFRvIII prevalence (20, 38). It is important to remember that HER2 amplification in HNSCC was initially considered rare and inconsistently detected, but later (87), emerged as an actionable target following rigorous validation and the availability of effective HER2-directed agents. This emphasizes that dismissing rare variants without definitive negative evidence may prematurely close therapeutic avenues; the absence of evidence is not evidence of absence (88), particularly when detection methods remain unstandardized. This indicates the need for further definitive validation studies employing state-of-the-art methods before abandoning EGFRvIII as a target in HNSCC. Rather than large-scale screening evidence, we ask for a targeted investigation in the contexts that merits exploration like cases with cetuximab refractory, chemo-resistant HNSCC patients.

In comparison with the GBM detection standards, there is a stark difference in EGFRvIII validation, as GBM had robust validation achieved through orthogonal detection platforms that cross validate one another with high concordance rates in quality controlled studies (66, 89). The infrastructure for standard sample processing including the GBM tissue banks by TCGA with rigorous quality control metrics (RNA integrity number >7), standard nucleic acid extraction protocols, development of real time RT-PCR assays for detection from FFPE samples (47) and systematic documentation of ischemic time has been largely absent in HNSCC cohorts (90). Multiple clinical trials were done to provide clinical relevance of EGFRvIII positive GBM (91).

The path forward demands methodological reproducibility and scientific rigor. Paradoxical correlations such as improved disease control in Chau et al.’s cohort underscore how technical artifacts can skew clinical interpretation (39). EGFRvIII-targeted strategies should be reserved for the rare patients whose tumors truly harbor this variant, confirmed through orthogonal, state-of-the-art detection methods. Only through such disciplined investigation can we avoid the twin perils of premature dismissal of a potentially actionable target and over-interpretation of technical artifacts in this molecularly diverse disease. The stakes are high as patients with rare oncogenic drivers deserve the same precision medicine advances as those with common alterations, but only if our detection methods and clinical validation are equal to the challenge.

## 8 Proposed consensus to be considered

To resolve controversies surrounding EGFRvIII detection in HNC, the field requires stringent consensus standards for future studies. We propose implementation of the guidelines required for GBM tissues: all putative EGFRvIII-positive cases must be confirmed using at least two independent detection platforms, including nucleic acid methods like digital droplet PCR (ddPCR) with 0.01% sensitivity or next-generation sequencing with a minimum 1000× coverage, plus protein-level confirmation through immunohistochemistry with validated EGFRvIII-specific antibodies. Sample quality control is essential, requiring RNA integrity numbers ≥7, a minimum 30% tumor cellularity, documented ischemic time under one hour, and at least 20 ng DNA input. Detection thresholds should be harmonized with a minimum variant allele frequency of 1% to distinguish true signals from background noise, alongside standardized reporting of all technical parameters including VAF (Variant Allele Frequency), tumor cellularity, and detection methods. Every experimental run must include both EGFRvIII-positive cell line controls and WT EGFR controls to validate assay performance.

Future studies should be prospective (39) rather than retrospective, with predefined detection protocols registered before patient enrollment and coordinated across at least three independent institutions to avoid site-specific artifacts and selection bias. Clinical validation requires clearly defined endpoints established prior, with overall survival and progression-free survival as primary endpoints for prognostic studies, while predictive studies should compare response rates between EGFRvIII-positive and negative subgroups. Therapeutic trials testing EGFRvIII-directed agents should enroll only patients with orthogonally confirmed positive tumors, using objective response rates and safety as primary endpoints with adequate statistical power calculations. This multi-platform concordance approach mirrors established practices in HER2 testing and addresses the technical artifacts that have plagued previous single-method studies. These rigorous standards, incorporating lessons from failed GBM vaccine trials and successful HPV biomarker validations, provide the necessary framework for definitive resolution of EGFRvIII’s role in HNSCC. A multi-phase consortium should establish standardized EGFRvIII detection protocols and conduct prospective studies (n=500-750) to definitively determine prevalence, clinical associations, and potential therapeutic relevance in HNSCC over 5–7 years. If prevalence is confirmed <1% without clinical significance, the field should formally abandon EGFRvIII as a biomarker and redirect resources to more promising targets.

## 9 Conclusion

As shown in this review of literature reporting EGFRvIII expression in HNSCC, early reports of high frequency, aggressive disease associations have given way to ultra-sensitive assays revealing the true rarity and questionable significance of EGFRvIII in this malignancy. To avoid perpetuating the confusion of the past two decades, the field needs to employ tools validated for this biomarker in other cancers like digital PCR, next-generation sequencing, standardized IHC, and multi-institutional collaborative networks to definitively answer whether EGFRvIII is a needle worth finding in the HNSCC haystack. What remains is the collective will to execute adequately powered, methodologically sound studies that will either validate EGFRvIII as a bona fide clinical target in HNSCC or close this chapter definitively. In summary, EGFRvIII in HNSCC teaches us that biology and methodology are inseparable and EGFRvIII-targeted strategies should be reserved for the rare patients whose tumors truly harbor this variant.
